# The Impact of a Graded Maximal Exercise Protocol on Exhaled Volatile Organic Compounds: A Pilot Study

**DOI:** 10.3390/molecules27020370

**Published:** 2022-01-07

**Authors:** Liam M. Heaney, Shuo Kang, Matthew A. Turner, Martin R. Lindley, C. L. Paul Thomas

**Affiliations:** 1 School of Sport, Exercise and Health Sciences, Loughborough University, Loughborough LE11 3TU, UK; m.r.lindley@lboro.ac.uk; 2 Centre for Analytical Science, Department of Chemistry, Loughborough University, Loughborough LE11 3TU, UK; julykang@hotmail.com (S.K.); m.a.turner@lboro.ac.uk (M.A.T.); c.l.p.thomas@lboro.ac.uk (C.L.P.T.); 3 Translational Chemical Biology Research Group, School of Sport, Exercise and Health Sciences, Loughborough University, Loughborough LE11 3TU, UK

**Keywords:** exhaled breath, VOCs, exercise, metabolomics, mass spectrometry

## Abstract

Exhaled volatile organic compounds (VOCs) are of interest due to their minimally invasive sampling procedure. Previous studies have investigated the impact of exercise, with evidence suggesting that breath VOCs reflect exercise-induced metabolic activity. However, these studies have yet to investigate the impact of maximal exercise to exhaustion on breath VOCs, which was the main aim of this study. Two-litre breath samples were collected onto thermal desorption tubes using a portable breath collection unit. Samples were collected pre-exercise, and at 10 and 60 min following a maximal exercise test (VO_2MAX_). Breath VOCs were analysed by thermal desorption-gas chromatography-mass spectrometry using a non-targeted approach. Data showed a tendency for reduced isoprene in samples at 10 min post-exercise, with a return to baseline by 60 min. However, inter-individual variation meant differences between baseline and 10 min could not be confirmed, although the 10 and 60 min timepoints were different (*p* = 0.041). In addition, baseline samples showed a tendency for both acetone and isoprene to be reduced in those with higher absolute VO_2MAX_ scores (mL(O_2_)/min), although with restricted statistical power. Baseline samples could not differentiate between relative VO_2MAX_ scores (mL(O_2_)/kg/min). In conclusion, these data support that isoprene levels are dynamic in response to exercise.

## 1. Introduction

Metabolomics has emerged to become a powerful tool within the bioanalytical locker through its capacity to provide information on the small-molecule metabolites that are present within the biological system and linked to physiological state [1]. These technologies have allowed researchers to expand the scope in which a biochemical profile can be constructed, with applications across a wide variety of topics including clinical and medical science [2], forensic investigations [3], food and nutrition [4] and sports science [1].

One subsection of metabolomics includes the measurement of volatile organic compounds (VOCs) present within exhaled breath gases [5]. These compounds, which are a major contributor to the total human volatilome [6], have been studied for their capacity to provide biochemical information from the human system using a none or minimally invasive collection protocol [7]. This approach has garnered particular interest within medical research owing to the potential for out-of-clinic collection, with a recent and topical interest shown in the capacity to diagnose COVID-19 disease through a single breath collection [8].

Although exhaled VOCs have been investigated within the sport and exercise remit, at present these studies remain limited and have predominantly reflected changes following low to medium intensity exercise, limiting their capacity to be translated to more competitive sporting scenarios [9]. It is, therefore, of interest to scientists in this area to understand if the data seen at low-grade exercise are translatable to higher intensities, and whether the exhaled VOC profile of individuals could be altered by physical fitness.

This exercise testing approach could be of further interest to the metabolomics community as many studies look to employ a ‘control’ group of participants. These participants are considered as fit, healthy, and free from disease. The consideration of the term ‘fit’, however, has an entirely different connotation when applied to the level of physical fitness of an individual. For example, a person could be described as fit and healthy by a physician, but in an exercise context their physical fitness could be markedly low. Consequently, this individual would not be considered as ‘fit’ by a sports scientist. For this reason, an improved understanding into the variability within a control group of participants would provide further insight into the applicability of its use within a clinical study, for example.

In this study, the collection of exhaled VOCs prior to and following exhaustive exercise was performed. The data were investigated for profile differences across these timepoints, as well as considering whether baseline (i.e., at rest) samples were able to identify differences in physical performance capabilities.

## 2. Results

### 2.1. Changes in Exhaled VOCs Following Exercise

**Figure 1:**
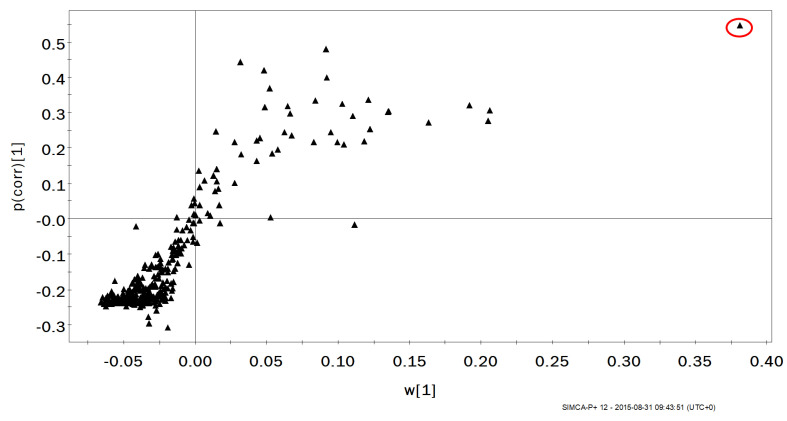
S-plot for modelled variables using an orthogonal partial least squares-discriminant analysis model for pre-exercise and post-exercise breath samples. A candidate biomarker is highlighted in the red oval.

**Figure 2:**
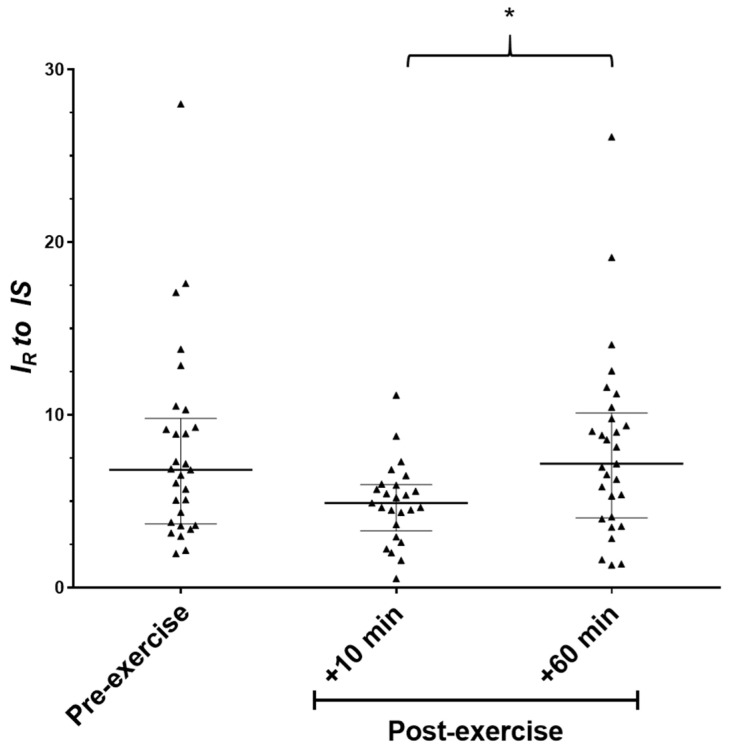
Box and whisker plots to display exhaled isoprene levels for all participants (*n* = 33) at pre- and post-exercise timepoints. * Denotes *p* = 0.041. Note: *I_R_* = relative intensity; IS = internal standard.

**Figure 3:**
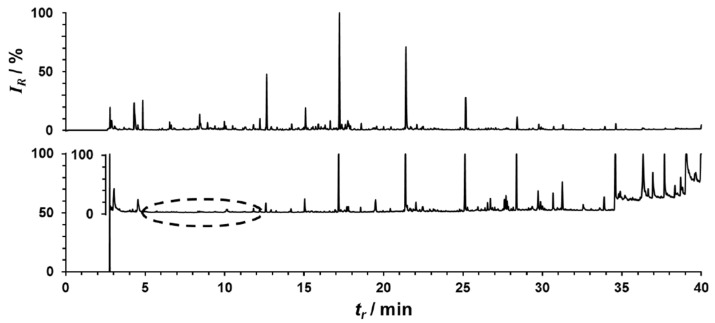
Example chromatograms to show an ejected breath sample due to excess solvent at 2.8 min (bottom) and a zoomed chromatogram for post-solvent peak responses (bottom inset) with a region of disrupted chromatography highlighted (dashed oval); and an acceptable exhaled breath sample from the same participant (middle). Note: *I_R_* = relative intensity; *t*_r_ = retention time.

To assess changes in VOCs caused by the maximal exercise protocol, multivariate analysis was performed across the three exercise stages. Initial analysis performed by OPLS-DA showed no separation in the 2D score plot. However, samples relating to the 10 min timepoint showed a partial separation from the pre and 60 min post-exercise timepoints. On analysis of the corresponding S-plot, only one VOC was observed to be providing a strong influence on the computed model (Figure 1). An investigation into the retention index (RI) and database searching (NIST Mass Spectral Database) indicated that the identified compound was 2-methyl-1,3-butadiene (isoprene). A Kruskal–Wallis H test indicated that there was not an equal distribution of ranks across the three timepoints and pairwise comparisons indicated an increase in exhaled isoprene between the 10 and 60 min post-exercise timepoints (*p* = 0.041). A reduced trend was observed for isoprene at 10 min post-exercise when compared to baseline levels, but statistical confidence was not reached. Similarly, no differences were observed between baseline values and 60 min post-exercise (*p* ≥ 0.269, Figure 2). Exhaled isoprene data were not available for all participants at all timepoints due to an overloading of the GC column with exhaled vapour/solvent in a small number of samples (see Figure 3); this may account in some way for the lack of power in this experiment.

### 2.2. Comparison of Upper and Lower Tertiles of Maximal Oxygen Uptake

OPLS-DA analysis reported no identifiable differences in exhaled VOC profiles when comparing the upper and lower tertiles of relative VO_2MAX_ values. Therefore, investigations for this factor were not continued further.

**Figure 4:**
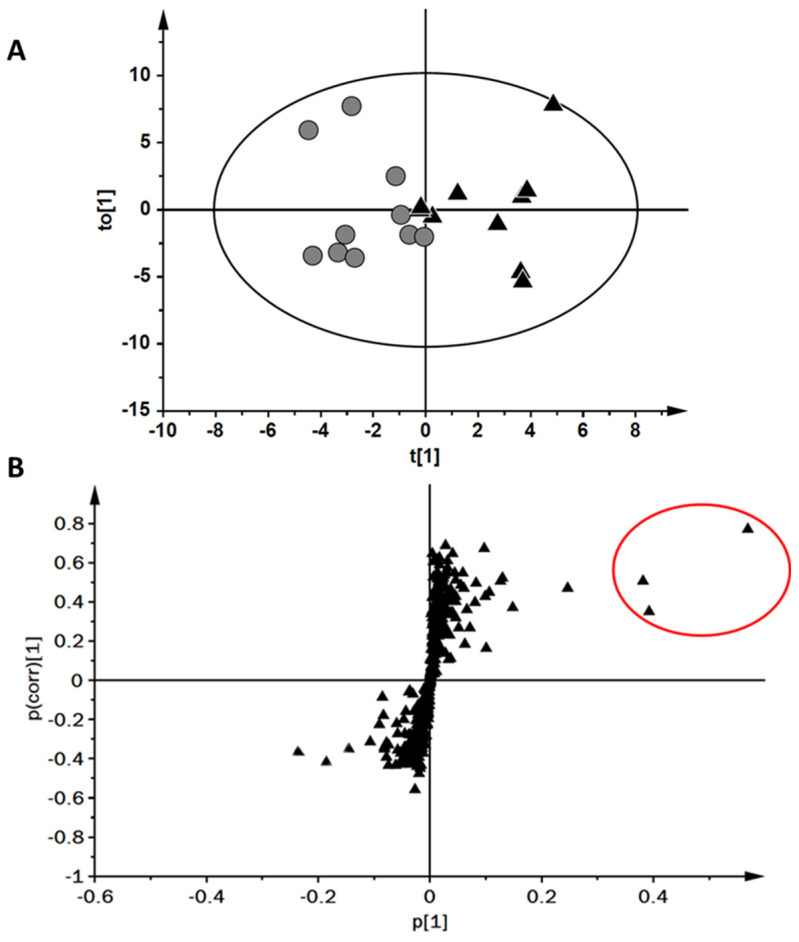
(**A**) Orthogonal partial least squares-discriminative analysis two-dimensional score plot constructed for breath samples for those falling into low (black triangles) and high (grey circles) absolute maximal oxygen uptake groups. (**B**) S-plot for modelled variables with candidate biomarkers highlighted in red ovals.

OPLS-DA analysis performed on high vs. low absolute VO_2MAX_ groups reported a complete separation of groups within the score plot (Figure 4A). The accompanying S-plot for the computed model was analysed and three upregulated regulated compounds were selected for further investigation (Figure 4B). The three upregulated compounds were evaluated against the VO_2MAX_ scores for the two stratified groups. Two features, when examined more closely, were identified as the same compound with altered ion statistics across samples (i.e., most abundant ion and secondary ion reversed). This information was used to combine these features. An investigation into the RI and database searching indicated that the two identified compounds were propan-2-one (acetone) and isoprene.

**Figure 5:**
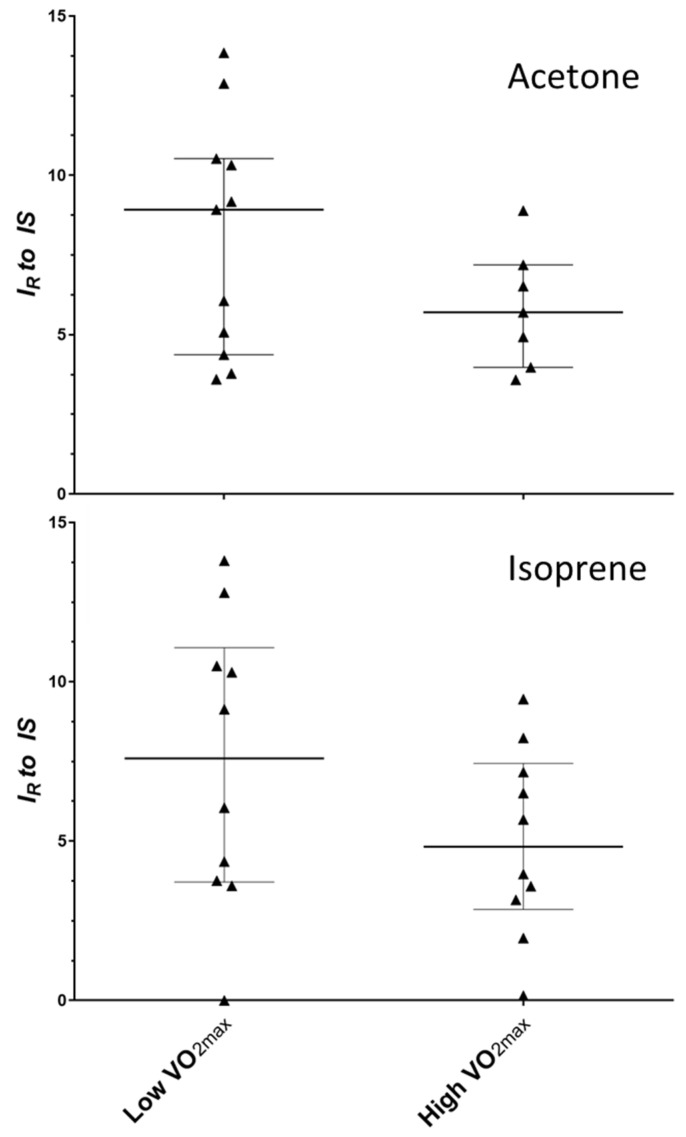
Plot to show the distribution of acetone (**top**) and isoprene (**bottom**) in low and high absolute VO_2MAX_ groups in a pre-exercise exhaled breath sample. Horizontal line is shown at the median with errors bars indicating the interquartile range. Note: *I_R_* = relative intensity; IS = internal standard.

Targeted inspection of the data from analytical peaks relating to acetone and isoprene showed the tendency for reduced levels of both compounds within the upper tertile grouping of absolute VO_2MAX_ scores (Figure 5); however, the large intra-group variation seen amongst participants meant that confident differences could not be confirmed (*p* = 0.247 and 0.190 for acetone and isoprene, respectively). 

## 3. Discussion

Non-targeted metabolomics-wide analysis of exhaled breath in physically fit individuals prior to an exercise capacity test was able to provide some indication of differences based on absolute maximal oxygen uptake figures. Breath metabolites of acetone and isoprene showed an observed reduction for individuals with elevated absolute maximal oxygen uptake (measured in L/min O_2_ consumption). However, a small sample size and high inter-individual variability did not allow for differences to be confirmed. No differences between groups were observed when a relative maximal oxygen uptake was assessed (normalised to body weight), and therefore the test did not show the capacity to predict the fitness of an individual from a breath test alone. The process of creating a test value relative to body mass may have led to the normalisation of metabolite values across the cohort.

Exhaled breath collections analysed prior to and in stages for up to one h post-exercise showed a fluctuation in exhaled isoprene levels. A reducing trend post-exercise was observed and returned to baseline values by one h, with the 10 and 60 min post-exercise timepoints reporting statistical differences. These data conform to previous on-line breath analysis which identified a rise in exhaled isoprene at the onset of exercise, followed by a reduction during the exercising period [10]. This was theorised to be due to the removal of an ‘isoprene store’ in the working muscles. When multiple exercise bouts were performed, a blunted increase in isoprene was seen at exercise onset without adequate recovery, and therefore supporting the idea that these stores were not rapidly replenished. The data presented in this study support this theory as a tendency for levels to be reduced was seen after a bout of intense physical activity, with a return to baseline following a sufficient rest time of one h. It is important to note that in this study, the impact of the exercise can be considered to be substantially higher than the previous study by King et al. [10]. This previous study used a low-grade cycling exercise of 75 W for 15 min. In comparison, the present study had an average exercise period of 19 min, similar to the previous work; however, the average final workload for the exercise was 276 W. This may account for the tendency for further reduced isoprene below baseline levels; however, further follow up studies are required to confirm these preliminary data.

Previous efforts into exercise-based research have generally included low-intensity and/or short duration, e.g., 75 W for 15 min [10] or have provided inadequate descriptions of the exercise protocol [11]. However, one study investigated the changes in exhaled breath content after a 1-h cycling time-trial, noting that 44 VOCs showed dynamic changes from pre- to post-exercise sample timepoints [12]. The increased number of discriminant VOCs seen in this previous study compared to the current investigation may be related to the duration of exercise performed. The authors were not able to confidently identify many of these ion traces, although increased levels of isoprene and acetone were observed post-exercise. Acetone and isoprene are perhaps the most extensively studied VOCs in exhaled breath. Acetone is produced endogenously via the decarboxylation of acetoacetate (a derivative of lipolysis) [13], with isoprene a metabolic by-product from the mevalonate pathway of cholesterol biosynthesis [14]. The reason for the potential reduction in these compounds in the high absolute VO_2MAX_ group is not known, with the increased BMI in this group perhaps suggesting the opposite might have been expected. One potential reason for this observation could be due to the format of exercise commonly completed within this group. The upper tertile showed an increased mean value for weekly vigorous exercise (*p* = 0.052) and decreased low intensity exercise (i.e., walking, *p* = 0.002). These differences in regular exercise intensities may cause alterations in acetone and isoprene production and/or storage. However, these exercise data are via a self-reported questionnaire and so should be considered carefully with the need for a more focussed interventional experiment required for confirmation.

The use of a control group is a necessity in many metabolomics-based experiments. Interestingly this cohort identified as fit, young, and healthy male participants would be considered a suitable control group. However, these data begin to highlight that within a relatively homogenous group, differences in exhaled profiles due to maximal oxygen uptake may be observed. In addition to this, the data suggest that changes in the routinely identified biomarker isoprene could be observed following exhaustive exercise. Whilst the exercise performed in this experiment is designed to push the participants to volitional fatigue, it must be considered in future that diseased patients may be working at high physical output levels during everyday activities (e.g., climbing stairs), and so these factors must be taking into consideration should these biomarkers be proposed for use in the clinic or for health monitoring.

In conclusion, the current study identified the potential for differences in exhaled VOC profiles within a homogenous group of young, fit and healthy men. These differences could not be confidently confirmed due to inter-person variability and therefore a larger study group is required. The test was not able to provide identification of physical fitness via a single exhaled breath sample at rest. Finally, the data support the previous identification that isoprene levels are dynamic in response to exercise, and the first time this has been completed for sorbent-captured VOC exhaled breath sampling using a high-intensity, maximal exercise protocol.

## 4. Materials and Methods

### 4.1. Ethical Clearance

This study was approved in its entirety by the Loughborough University Ethical (Human Participants) Sub-Committee. All participants took part voluntarily and were informed of the experimental procedures by issue of a participant information sheet prior to consenting. All participants gave written and informed consent and were free to exclude themselves and their data from the experiment at any time without reason. Once consented, participant information and samples were anonymised and assigned a unique identifier code.

### 4.2. Participant Information

Thirty-three healthy males (mean ± standard deviation: age 23 ± 3 years, height 180 ± 6 cm, body mass 82.2 ± 10.3 kg, body mass index (BMI) 25.3 ± 2.4 kg/m^2^) were recruited and completed the research protocol. All the participants reported active engagement in sporting behaviours at either an individual or team level. All participants were free from injury.

### 4.3. Experimental Design

**Figure 6:**
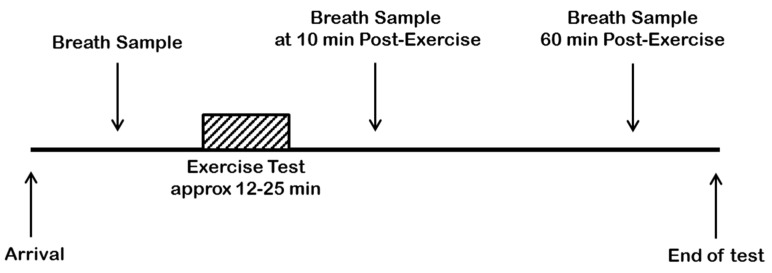
A schematic diagram to show the experimental protocol for each participant from arrival at the laboratory until cessation of the final exhaled breath sample.

Participants arrived at the laboratory at approximately 830 h following an overnight fast, with only water intake permitted after waking and until the cessation of the study visit. On arrival, each participant was asked to sit quietly for five min before the test protocol started. The test protocol consisted of providing a resting exhaled breath sample, completing a graded maximal oxygen uptake (VO_2MAX_) test, with two further exhaled breath sample collections at 10 and 60 min post-exercise. A schematic diagram to visualise the experimental design is shown in Figure 6.

### 4.4. Exercise Protocol

An incremental, steady state, exercise protocol was performed using an electromagnetically braked cycle ergometer (Lode Excalibur Sport, Lode B.V., Groningen, The Netherlands). Exercise intensity began with a work output of 95 W, increasing in 35 W steps every 3 min. No prior warm up was performed as the low intensity exercise in the principal three stages was deemed light enough to suitably prepare the lower limb muscles for the more intense stages. Total exhaled gases were collected in an evacuated Douglas bag in the final min of each 3 min stage and later analysed for O_2_ and CO_2_ content. The participants were also asked to rate their perceived level of exertion during each breath collection by pointing to a value on the Borg RPE scale [15]. The participants were instructed to inform the researcher when they had one min of effort remaining and a final exhaled gas collection was performed and used to calculate VO_2MAX_ as both a relative (normalised to body weight) and absolute value.

### 4.5. Exercise Testing

All participants completed the exercise test to volitional fatigue. The common protocol for the successful attainment of maximal oxygen uptake (VO_2MAX_) include a measurement of heart rate (HR) within 10 beats/min of predicted maximum ($HR_{\left(max\right)}=220-age\;in\;years$) and a respiratory exchange ratio (RER) of ≥1.1 [16]. It is expected that participants would report a rating of perceived exhaustion (RPE) [15] of ≥19 within the final min of exercise. Fifteen of the 33 participants satisfied these criteria, with the remainder of participants satisfying at least one criterion.

Two participants were subsequently excluded from the data analysis when their pre-exercise exhaled breath samples were discovered to contain levels of solvent molecules high enough to disrupt chromatographic behaviour.

An average relative (i.e., normalised to body mass) VO_2MAX_ score of 46.5 mL(O_2_)/kg/min was obtained with a range of 35.3–58.9 mL/kg/min. Group differences stratified by upper and lower tertiles (*n* = 10) of relative and absolute (i.e., without normalisation) VO_2MAX_ are displayed in Table 1. The high relative VO_2MAX_ group had decreased mass, height and BMI (≤0.023). No differences were seen between relative VO_2MAX_ groups and exercise test duration. The high absolute group had increased mass and BMI and performed less self-reported min of walking per week (*p* ≤ 0.005). The high absolute group were able to complete a longer period of exercise (*p* < 0.0005) during the maximal exercise test. No differences were seen for laboratory exercise conditions.

### 4.6. Exhaled Breath Sampling and Analysis

Exhaled breath VOCs were collected using a previously described portable breath collection unit [17]. Two litres of breath were sampled and VOCs retained onto an adsorbent bed packed into thermal desorption tubes (Markes International, Llantrisant, UK). Samples were analysed for VOC content by thermal desorption-gas chromatography-mass spectrometry (TD–GC–MS) using a Unity TD Unit (Markes International, Llantrisant, UK) coupled to Varian 3800 GC and Varian 4000 ion trap MS instrument (now Agilent Technologies, Stockport, UK). Detailed information on the analytical protocol can be found elsewhere [17,18].

### 4.7. Statistical Analyses

GC–MS data were exported from the Varian operating system and peak deconvolution was performed by AnalyzerPro (SpectralWorks, Runcorn, UK). Each VOC peak identified from deconvoluted data was assigned a unique identifier, and peak integration parameters were optimized for each candidate compound enabling peak integration data to be exported to an exhaled VOC breath matrix identified by retention index and main diagnostic ions. This information was used to produce a search function that prospected all samples, with 373 distinct peaks isolated, integrated and normalised to a post-loaded toluene-d8 internal standard.

Sample group or timepoint pairings were analysed by orthogonal partial least squares-discriminant analysis (OPLS-DA) to prospect for discriminating factors between groupings. S-plots were analysed for contributor variables. Discriminant components were assessed as potential biomarkers of exercise response by comparing whole group changes across timepoints, or biomarkers of fitness by comparing groupings of the upper and lower tertiles of VO_2MAX_ scores. 

Further data analyses were performed using IBM SPSS Statistics (v 22.0, IBM Corp., Endicott, NY, USA). Differences observed for isolated biomarkers of high and low groupings of VO_2MAX_ were assessed using the Mann–Whitney U test for independent samples. The distribution of exhaled VOC values observed across time were assessed using the Kruskal–Wallis H test for related samples. An alpha value (*p*) of <0.05 was deemed as statistically significant and, where appropriate, is reported as its Bonferroni-adjusted value.

## Figures and Tables

**Table 1:** Baseline and exercise characteristics of participants and environmental conditions grouped by upper and lower tertiles for relative and absolute maximal oxygen uptake (VO_2MAX_).

		Relative VO_2MAX_			Absolute VO_2MAX_		
		High	Low	*p* Value	High	Low	*p* Value
	Age (years)	23 (3)	22 (3)	0.247	24 (4)	22 (2)	0.796
	Mass (kg)	72.6 (6.2)	89.3 (9.1)	<0.0005	90.2 (9.6)	75.7 (9.2)	0.005
	Height (cm)	176 (7)	183 (5)	0.023	184 (6)	177 (7)	0.075
	BMI (kg/m^2^_)_	23.5 (1.4)	26.6 (2.4)	0.004	26.7 (2.0)	24 (1.5)	0.004
Weekly activity	Vigorous (min)	325 (196)	230 (113)	0.353	374 (173)	228 (152)	0.052
	Moderate (min)	89 (112)	144 (103)	0.143	174 (155)	168 (325)	0.218
	Walking (min)	225 (99)	292 (179)	0.280	133 (84)	331 (145)	0.002
	Relative VO_2MAX_ (mL(O_2_)/kg/min)	51.9 (2.6)	40.9 (2.8)	<0.0005	47.3 (5.9)	45.0 (5.6)	0.481
	Absolute VO_2MAX_ (L(O_2_)/min)	3.8 (0.4)	3.6 (0.4)	0.481	4.2 (0.2)	3.4 (0.1)	<0.0005
	Final stage heart rate (beats/min)	191 (7)	186 (9)	0.277	185 (10)	192 (5)	0.063
	Room temperature (°C)	21.1 (1.0)	21.4 (0.8)	0.247	21.1 (0.9)	21.2 (1.1)	0.796
	Total exercise time (min)	19 (3)	18 (2)	0.387	20 (2)	17 (2)	<0.0005
	Room pressure (mmHg)	764 (7)	760 (6)	0.218	762 (14)	764 (6)	0.353
	$F_{I}O_{2}$ (%)	21.0 (0.1)	20.9 (0.1)	0.165	21.0 (0.1)	21.0 (0.1)	0.631
	$F_{I}{\mathrm{CO}}_{2}$ (%)	0.05 (0.01)	0.04 (0.01)	0.739	0.04 (0.01)	0.04 (0.01)	0.971

Note: BMI = body mass index; CO_2_ = carbon dioxide; O_2_ = oxygen; $F_{I}$ = fractional inhaled. All data are expressed as the mean (standard deviation); *n* = 10 for both groups.

## Data Availability

The data presented in this study are openly available in FigShare at https://doi.org/10.17028/rd.lboro.17074841 (accessed on 25 November 2021).

## Sample Availability

No samples from this study are available due to the destructive nature of the analytical protocols.
